# Exposure to Harmful Dusts on Fully Powered Longwall Coal Mines in Poland

**DOI:** 10.3390/ijerph15091846

**Published:** 2018-08-27

**Authors:** Jarosław Brodny, Magdalena Tutak

**Affiliations:** 1 Faculty of Organization and Management, Silesian University of Technology, 41-800 Zabrze, Poland; jaroslaw.brodny@polsl.pl; 2 Faculty of Mining and Geology, Silesian University of Technology, 44-100 Gliwice, Poland

**Keywords:** harmful dusts, occupational safety and health, mining exploitation, crystalline silica, occupational diseases

## Abstract

The mining production process is exposed to a series of different hazards. One of them is the accumulation of dust which can pose a serious threat to the life and health of mine workers. The analysis of dust hazard in hard coal mining should include two aspects. One is the risk of coal dust explosions, which poses a direct risk of injury or even loss of life, the second is the risk of harmful dust, associated with the possibility of negative health effects as a result of long-term exposure to dust in the worker’s body. The technologies currently applied in underground mining produce large amounts of coal and stone dust. Long-term exposure to dust and crystalline silica may cause chronic respiratory disease. The article presents the results of tests on the dust levels in the area of a fully-powered longwall. The tests were conducted for five longwalls from different hard coal mines. In each of them, the average values of inhalable and respirable dust as well as the percentage content of free silica in the dust were determined in ten selected working positions. Additionally, for the longwall with the highest dust concentration, the levels of dust were determined for the basic activities related to the phases of the technological cycle. The comparative analysis conducted and the results obtained demonstrate large variations in the dust levels in the different areas. The permissible values were significantly exceeded in a number of cases. This poses a great threat to the health of Polish miners. The results obtained indicate that it is necessary to undertake more effective measures in order to improve the working environment of the crew in hard coal mines.

## 1. Introduction

The mining production process is accompanied by a series of different hazards. They constitute a significant source of risks and may pose a threat to the life and health of employees and large material losses. Risk is the chance or probability that a person will be harmed or experience an adverse health effect if exposed to a hazard [1], while a hazard is any source of potential damage, harm or adverse health effects to something or someone under certain conditions at work. Basically, a hazard can cause harm or adverse effects (to individuals as health effects or to organizations as property or equipment losses) [1].

For this reason, one of the most important tasks during this process is to ensure adequate levels of safety for mine workers. Depending on the type of hazard, relevant steps are taken to reduce the likelihood or consequences of its occurrence. The presence of hazards in underground mining is related to the ongoing exploitation activity and results from the disruption of the initial balance in the intact rock mass. One of such hazards that poses a significant threat to the life and health of mine workers is harmful dust [2,3,4,5,6]. It results from the widespread presence of coal and stone dust in mine headings (as a mixture of silica, aluminosilicates and other elements, including trace metals), which is generated in the mining and transportation of the excavated coal material [2,3,4,6].

The dust formed during the mining of the body of coal and the dredging of dog headings, fills the mining atmosphere and travels through the ventilation system into most of the mine headings, including those located far away from the source, thereby causing their contamination [7,8,9].

It is estimated that the amount of dust generated may be as much as approx. 3% of the mass of the whole excavated coal material [2,10]. Assuming that the average daily mining output in a mine is approximately 10,000 Mg, the quantity of dust generated may amount to approx. 300 kg/day.

The quantity of dust generated during exploitation depends on a number of factors, which can be divided into mining/technical and geological [11,12,13,14,15,16,17,18]. The former include the manner in which the rock mass is mined (including the type of the cutting machine), the type and condition of the cutting knives, the type and condition of the sprinkling system, as well as the speed of exploitation (including the speed of cutting), the thickness of the seam exploited [11,12,13].

The concentration of dust that rises in mining excavations is influenced by such factors as the velocity of air flowing through the excavations (high velocities can carry particles of dust previously settled on the floor, side walls and devices) and the number of free surfaces in the wall cross-section [7].

The second one includes the type of rock mined and its mineralogical composition, cleavage, compactness (easily mineable coals cause greater dusting than coals with higher mineability rates), hardness, volatile matter content in the coal (the higher this content in coal, the higher dust concentrations are generated during the mining process) and the ash content in the coal. Increased ash content leads to increased total dusting. However, it does not result in higher concentrations of the respirable fraction that is primarily responsible for the detrimental effects of dust on the human body. This results from the physical structure of coal components that form the ash and the coal moisture content (dust levels are lower when the moistness of the seam ranges from 0 to 10%, yet increase when the moistness exceeds 10%) [14,15,16,17,18].

The specific risk for the health of workers exposed to the impact of dust arising from the exploitation of hard coal is created by the free crystalline silica contained in this dust. The specific risk for the health of workers exposed to the impact of dust arising from the exploitation of hard coal is created by the free crystalline silica contained in this dust [19,20,21,22]. Prolonged work in a high-dust environment, where dust levels exceed the permissible values and where, additionally, the free crystalline silica fraction is present, may cause serious lung diseases (mainly pneumoconiosis) in miners.

This condition is the most commonly reported occupational disease amongst current and former hard coal miners, not only in Poland but also in China and the USA. According to the statistics released by the Ministry of Health in China, more than 300,000 coal miners suffered from pneumoconiosis by the end of 2007, accounting for 50% of the total number of pneumoconiosis patients in China. Every year, more than 10,000 people who work in major state-owned coal mines are added to the list of pneumoconiosis patients, and on an average, 2500 Chinese miners die from this disease [4]. 

In the USA a gradual increase in the prevalence of pneumoconiosis amongst miners has also been recorded since the late 1990s. The most disconcerting trends have been observed in parts of central Appalachia (e.g., MSHA districts 4 and 12), and many new cases of CWP (pneumoconiosis-CWP, sometimes referred to as “black lung”) and/or silicosis appear to be advanced or presenting in younger miners [23].

**Figure 1:**
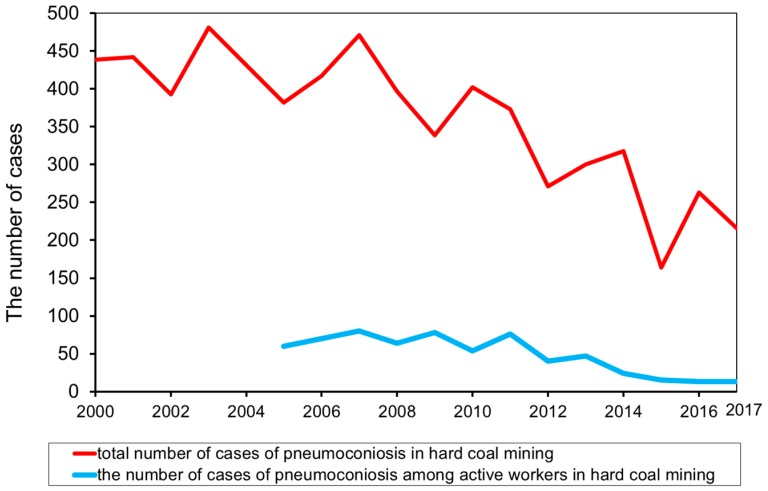
The number of reported cases of pneumoconiosis among workers of hard coal mines in Poland in 2000–2017.

In Poland, in the years 2000–2017, there was a total of as many as 7340 diagnosed cases of pneumoconiosis amongst current and former mine workers [24]. A comparison of the number of cases of pneumoconiosis among workers of hard coal mines is presented in Figure 1. The number of cases of active mine employees has also been taken into account (this is due to the data available) since 2005. It should also be emphasized that 80% of diagnosed cases of pneumoconiosis in Poland referred to active and former employees of hard coal mines. The proportions have remained at a similar level for many years. At the same time, it should be emphasized that the pneumoconiosis accounts for as much as 76.6% of all occupational diseases among miners of Polish hard coal mines [24].

Pneumoconiosis is an incurable diseases which can still progress even after cessation of harmful dust exposure. A crucial impact on the development of this disease is exerted by the particle size and the type of dust inhaled. Irreversible health changes, on the other hand, are caused by respirable dust penetrating into the human body. Respirable dust is the fraction of airborne particulates that can be deposited anywhere in the lung gas-exchange region [25].

The dusts affecting the human body can generally be divided into collagen and non-collagen dusts. The former ones show biological activity causing focal fibrosis of the pulmonary tissue through their toxic effect on the macrophages. The latter are characterised by the fact that they accumulate only in the pulmonary alveoli, without causing permanent changes in the body. Irreversible changes caused by pneumoconiosis in the pulmonary alveoli represent the process of phagocytosis in which the collagen dust contributes to the decay of macrophages (the connective tissue cells). These dead cells form deposits which become fibrous over time and thus decrease the surface area of the pulmonary alveoli. They are replaced by newly-formed phagocytes. The process whereby irreversible lesions are becoming noticeable takes from a few to a dozen or so years and may exhibit a mild or severe course. It takes approximately 10–20 years for the lesions caused by mild simple pneumoconiosis to become visible, whereas severe pneumoconiosis, or the so-called silicosis, does not manifest itself until after approximately 5–10 years of exposure to harmful dust [8,9,14,26,27,28,29].

Pneumoconiosis in coal mine workers represents approximately 81% of all the registered cases of this disease in the Polish industry [24]. These unfavourable statistics lead to a series of measures being taken in mines in order to reduce the workers’ exposure to dusts. Nevertheless, it is not uncommon, in practice, to record dust concentrations in the mine atmosphere which significantly exceed the permissible values. This is because it turns out that complete elimination of the harmful dust hazard in hard coal mining is currently not possible. The reason for this is that these dusts are the by-product of polydispersive processes and it is impossible to completely eradicate their formation with the technology currently in use. 

Therefore, it is reasonable to conduct tests and analyses with a view to diagnosing the current levels of dust in mines, as well as to develop guidelines concerning the improvement of this situation.

The issues related to the dust hazard appear in numerous publications [2,3,4,5,6,7,11,12,15,16,23,30,31,32,33], but they mainly concern the methods for measuring dust levels in mine headings [2,4,11,30], the analysis of average dust values occurring in underground mining or for the particular groups of working positions [31,32,33]. As a result, the publications mostly provide a general analysis of the dust content in all the mine headings, without account being taken of the type of these headings (longwall or dog headings) and the specificity of the particular working positions. By adopting the average dust levels for the purposes of analysis, these publications make it possible to illustrate merely the general scale of the problem and are primarily of a demonstrative nature. Such an approach, however, does not reflect the actual levels of dust in the particular working positions, which are—to a large extent—location-dependent.

Therefore, it can be concluded that the presentation of differences in dustiness harmful to health occurring at the same workplace, and located in different walls, is a new approach to the problem of this threat for workers in the Polish hard coal mining industry.

Taking into account the current state of knowledge and the serious hazard to human life and health caused by dust, the authors decided to conduct tests aimed at determining the concentration levels of harmful dusts in selected working positions in hard coal mines. The tests were carried out in five mines (for five different longwalls), in ten selected working positions located in the area of longwalls, i.e., in places which were assumed to exhibit the highest concentration levels of harmful dusts. 

The purpose of the tests and of the analysis conducted was to determine the actual concentration values of mining dusts in these working positions, taking into account the differences related to their location. Additional tests and analyses were performed in the working positions located in the area of the longwall with the highest dust levels. In this case, account was also taken of the activities performed by the workers during a single routine working shift which lasts 7.5 h.

The Authors related the results obtained to the regulations applicable in Poland concerning the Maximum Admissible Concentration (MAC) of dust containing free crystalline silica [34]. In the authors’ opinion, the manner of assessing the harmful dust hazard in Polish coal mines, as presented in the paper, should be used for taking more effective prevention measures and minimising the consequences of this hazard. The related activities should therefore represent one of the pillars in the responsible and sustainable development of this industry.

## 2. Materials and Methods—Dust Monitoring

**Figure 2:**
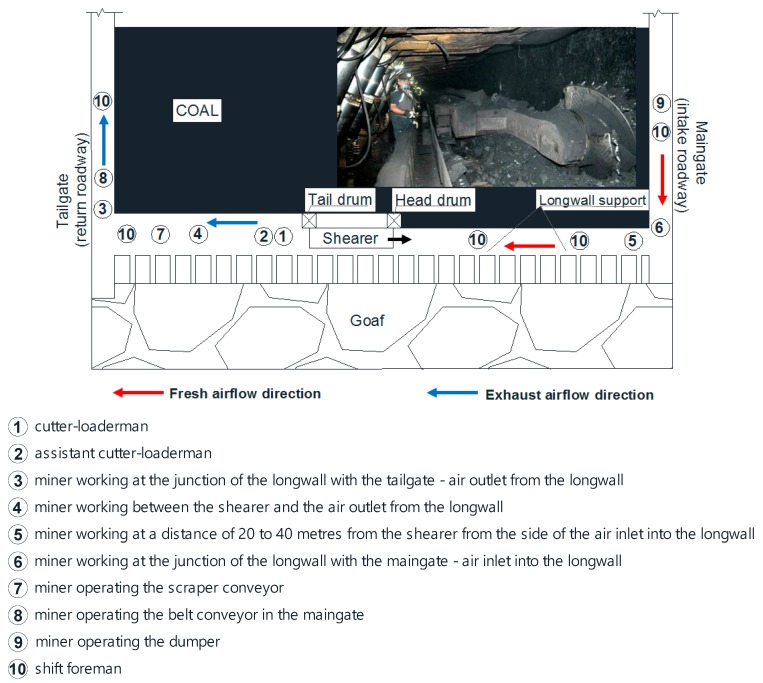
Typical U ventilation system in Poland with markers workplaces (modified from [35]).

The tests of dust levels were conducted in five hard coal mines (with one longwall analysed in each) located in the Upper Silesian Coal Basin in ten working positions. In these mines, exploitation is carried out by means of an automated longwall system. The longwalls under analysis are mined by means of longwall shearers. All the longwalls were ventilated with the U-type system (Figure 2).

Measurements of dust concentration occurring at workplaces were carried out in longwalls run with a system based on caving of roof rocks into the empty space left after extracted coal (goaf), in the case of filling the empty space with other material, however, we deal with backfill.

The measurements in each of the mines were conducted in the following working positions located in the area of the longwall and its adjacent headings: cutter-loaderman (1), assistant cutter-loaderman (2), miner working at the junction of the longwall with the tailgate—air outlet from the longwall (3), miner working between the shearer and the air outlet from the longwall (4), miner working at a distance of 20 to 40 m from the shearer from the side of the air inlet into the longwall (5), miner working at the junction of the longwall with the maingate—air inlet into the longwall (6), miner operating the scraper conveyor (7), miner operating the belt conveyor in the maingate (8), miner operating the dumper (9) and shift foreman (10). The distribution of these positions in the area under analysis, with account being taken of the indications placed in brackets (in the form of consecutive numbers), has been presented in Figure 2. The shift foreman (marked as 10) has no permanent working zone in the heading since his obligations encompass the supervision of ongoing works in the entire longwall. Hence, it is the most mobile working position which has been marked in a few places in the figure.

The selected workstations are typical for longwall mining. Between two or three employees usually work in one site, with the exception of a longwall shearer, a combine harvester assistant and a shift foreman. Measurements were made for individual employees representing a given workstations.

**Figure 3:**
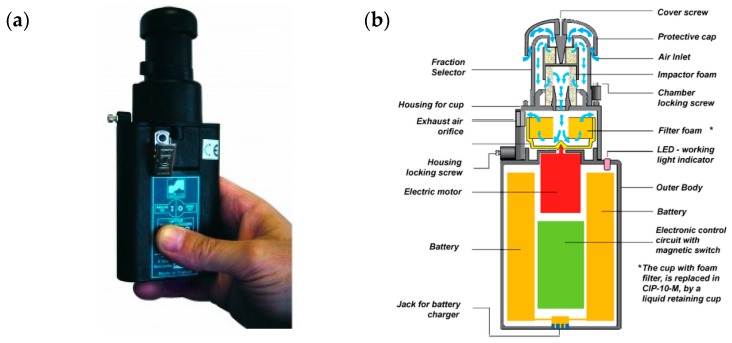
Personal dust sampler (**a**) [36] and schematic of CIP-10 Sampler (**b**) [37].

The measurements of dust levels were carried out by means of CIP-10-type personal dust samplers (Arelco ARC, Fontenay Sous Bois, France) using the dosimetric-individual method (Figure 3a). Schematic of CIP-10 Sampler has been presented in Figure 3b.

A CIP-10-type dust sampler is a gravimetric dust meter intended for assessing individual exposure to dust in the working environment. It measures the mass of dust in air. For a dust sampler to be selected for the tests, it had to be admitted to operation in places where explosive atmospheres may occur. The measurement error of the device used for the tests was ±0.05 mg/m^3^. The measurement error for dust concentration for stable airflow did not exceed 3% of the measured value [38]. The content of free crystalline silica in the dust was determined on the basis of the samples collected. The measurement time encompassed one working shift (7.5 h for air temperature up to 28 °C).

In each of the longwalls, the measurement of dustiness at a given workstation included measurements of inhaled (total) and respirable fraction (dust penetrating the alveoli). Then, for the collected samples, the content of crystalline silica was determined under laboratory conditions.

Measurements were carried out for three phases of the technological cycle (access to the workstation and preparatory work, mining of coal with a combine harvester and work carried out during the technological break—no mining). The duration of the measurement series during the implementation of the individual phases of the cycle ranged from 1.5 h (access to the workstation, preparatory work in the longwall) to 3 h (mining). No disturbances in the measurements took place in any of the five longwalls during tests.

Once the tests were completed, the dust samples collected on the filter placed in the CIP-10 dust sampler were weighed. For the selector dust to calculate the dust concentration is collected on the foam filter. Calculation of dust concentration *X_resp_* (mg/m^3^) is performed according to the following equation [2]:

(1) $$X_{resp}=\frac{\Delta m}{vt}$$

where: Δ*m* is mass of the coal dust collected on filter, *v* is the flow of air through the dust sampler (for CIP-10 it is 10 L/min) and *t* is measurement time (min).

Based on the measurement of the dust mass and the length of the dust samplers’ exposure to the dusty air, weighted average values were determined for the concentrations of total dust and respirable dust for the measurement period.

The preparation of the results involved determination of the weighted average values of the inhalable and respirable dust concentrations, the content of free crystalline silica in the dust and the exceedance rate of the MAC values (according to Polish regulations) for each of the working position under analysis [34].

Determination of free crystalline silica in total and respirable dust at workplaces is performed using the colorimetric method in compliance with the Polish standard [39]. The first phase is based on changing the crystalline free silica contained in the dust sample into soluble sodium silicate and then conducting colorimetric determination of silicate ions. This method of determination of silica in dust is currently used by the majority of laboratories in Poland [40]. Additionally, average values of the inhalable and respirable fraction concentrations were determined for each of the longwall areas under measurement, along with standard deviations and their medians.

## 3. Results and Discussion

**Figure 4:**
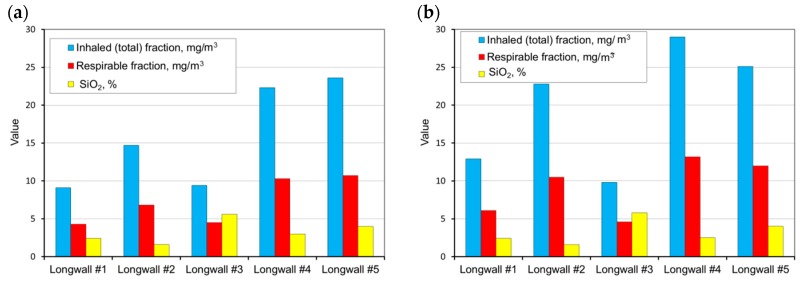
The measurement results for the dust levels in the positions of the cutter-loader man (**a**) and the miner working between the shearer and the air outlet from the longwall (**b**) (workplaces 1 and 4).

**Figure 5:**
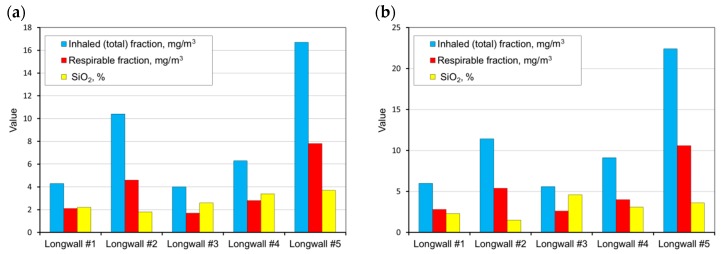
The measurement results for the dust levels in the positions of the miner working at the junction of the longwall with the maingate (air inlet) (**a**) and the shift foreman (**b**) (workplaces 6 and 10).

**Figure 6:**
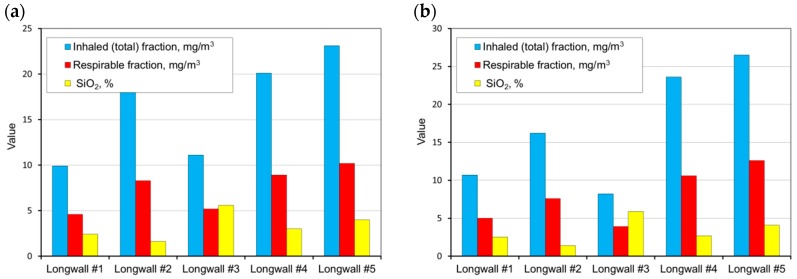
The measurement results for the dust levels in the positions of the assistant cutter-loader man (**a**) and the miner working at the junction of the longwall with the tailgate (air outlet) (**b**) (workplaces 2 and 3).

**Figure 7:**
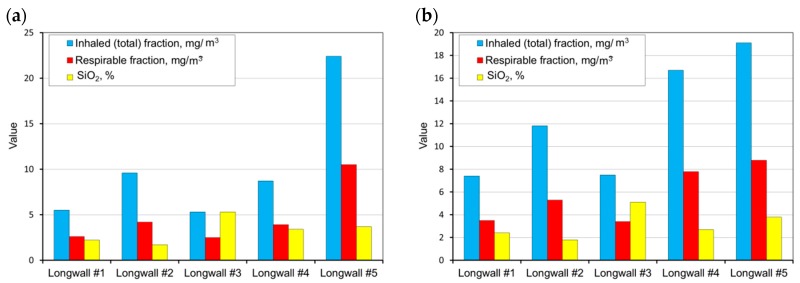
The measurement results for the dust levels in the positions of the miner working at a distance of 20 to 40 m from the shearer from the side of the air inlet into the longwall (**a**) and the miner operating the scraper conveyor (**b**) (workplaces 5 and 7).

**Figure 8:**
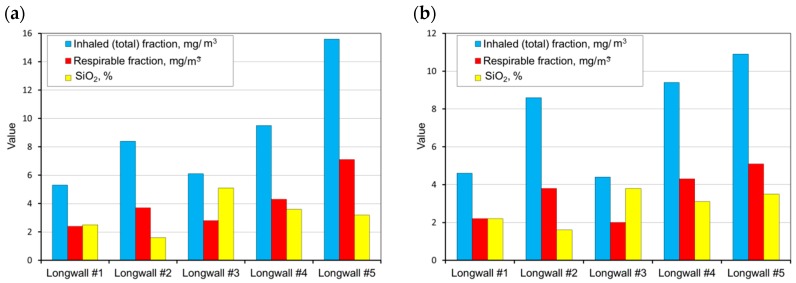
The measurement results for dust levels in the positions of a miner operating the belt conveyor (**a**) and a miner operating the dumper PZ/PT (**b**) (workplaces 8 and 9).

The measurements carried out were then used as a basis and allowed the determination of the average weighted total and respirable dust concentrations occurring at the tested sites and the content of free crystalline silica in this dust on the basis of the measurements carried out. These values were determined for all workstations in the tested longwalls (in each mine). A summary of the results obtained is presented in Figure 4, Figure 5, Figure 6, Figure 7 and Figure 8.

Analysing the results obtained, one can conclude that the highest dust values were registered in the following working positions:

– the miner working between the shearer and the outlet of air from the longwall,
– miner working at the junction of the longwall with the tailgate—air outlet from the longwall,
– the assistant cutter-loader man,
– the cutter-loader man.

The lowest dust levels were registered in the position of the miner operating the dumper. This is due to the location of this position in relation to the extracting machine and the manner of longwall ventilation. This position is located not in the longwall itself but in the maingate, along which fresh air is supplied to the longwall. The dust generated during the operation of a shearer has difficulties in getting through to this region. However, it finds it easy to travel along with the stream of fresh air into the opposite heading (the tailgate).

**Figure 9:**
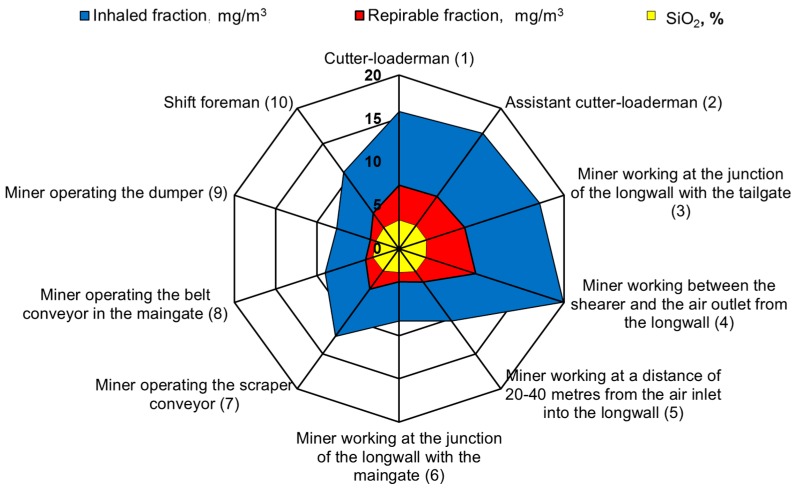
The average concentration of inhaled and respirable dust and the content of crystalline silica in various workstations.

The average concentration of inhaled and respirable dust as well as the content of crystalline silica in the various workstations in the walls are presented in Figure 9.

The measurement of dust levels in the working positions under analysis in the five longwalls made it possible to determine the average values of the inhalable and respirable fractions for these mines (Table 1). The mean content of free crystalline silica in the tested dust was also calculated (Table 2). The value was determined in a percentage as required by current Polish regulations. Table 2 presents average percentages of free crystalline silica for sites located in the subject longwalls.

**Figure 10:**
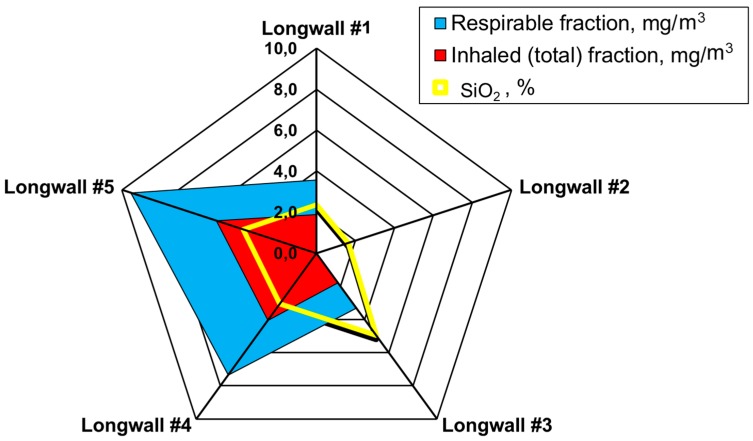
The average exceedance rate of the MAC values for dust in the longwalls under examination.

Based on the test results obtained, it can be concluded that the highest levels of dust occur in longwall no. 5. The workers of this coal mine have the highest exposure to the harmful effects of dust (the average value for the inhalable dust is higher than 20.0 mg/m^3^, and for the respirable dust—higher than 5.0 mg/m^3^). The lowest exposure to dust occurs in the area of longwall no. 1 and no. 3 (the average value for the dust inhaled is slightly higher than 7.0 mg/m^3^, and for the respirable dust—3.0 mg/m^3^). The values of concentrations admissible in Poland of harmful dust containing free crystalline silica are presented in Table 3. These values were used to determine the fold of the MAC exceeded level of the total and respirable dust in the tested longwalls (Figure 10).

The tests registered significant differences in the dust concentration values and in the percentage content of free silica during the performance of the particular activities related to the extraction cycle. These differences were dependent on the working position covered by the measurement. To illustrate these differences, an additional analysis was performed on the data for longwall no. 5 with the highest dust levels. These data were related to the activities (closely linked to the technological cycle) performed by the particular workers. They are characteristic of each working position covered by the measurement.

Implementation of the technological cycle in the exploitation wall includes the performance of a number of activities depending on the workplace, for example, works related to the mining of the combine by the combine harvester and assistant. Reconstruction of the intersections of the fail and head gates is conducted by the employees working at the inlet and outlet to the longwall. Moving the beam stage loader or the shortening of the belt conveyor are tasks performed by the miners servicing the scraper and wall conveyors. These are the main activities conducted simultaneously with the coal mining process.

When the combine moves along the wall by mining the coal, its movement should not be stopped by, for example, moving the support or other mining work. The only exception are the events resulting from the selected mining technology. This is the case, for example, when the main drive or return drive is moved. In this case, the longwall shearer is stopped for the duration of these activities. These are referred to as mining work carried out during the technological break.

The dust levels in the working positions in question were measured and analysed during three phases of the technological cycle. These phases encompassed the following:

– reaching the workplaces and preparatory works before the exploitation process;
– cutting of the body of coal by means of a longwall shearer;
– mining works during a maintenance shutdown.

The results obtained are presented in Table 4, Table 5, Table 6, Table 7, Table 8, Table 9, Table 10, Table 11, Table 12 and Table 13.

The results demonstrate that the location of a given working position along with the type of activities performed have a significant impact on the levels of dust. The lowest dust concentration levels were recorded in the position of the miner operating the dumper whereas the highest—in the working position located at the junction of the longwall with the tailgate (outlet). The low dust concentration levels in the position involving the operation of the dumper are due to the fact that the dumper is located at a considerable distance from the initial, direct source of the dust and lies in the inlet stream of air (with lower dust levels). Minor differences in dust levels are present in the positions of the miner working at a distance of 20 to 40 m from the shearer from the side of the air inlet into the longwall, the miner working at the junction of the longwall with the tailgate (air outlet), the cutter-loader man, the assistant cutter-loader man and the miner working between the shearer and the air outlet from the longwall. This is caused by the location of these positions in a close distance to each other and the performance of similar activities.

The smallest differences in dust concentration levels during a mining cycle and a maintenance shutdown were recorded in the positions located at the air inlet to the longwall. This is closely related to the direction of the airflow in this heading. The greatest differences occur, on the other hand, between the positions located at the air inlet and outlet points of the longwall.

## 4. Conclusions

The article presents the results of the tests and analyses concerning the concentration levels of dust and the content of free crystalline silica in the dust, as present in selected working locations in five Polish hard coal mines. The testing methodology applied made it possible to determine these values and conduct a comparative analysis. The results obtained unambiguously indicate that underground mining exploitation is characterised by extremely difficult working conditions in terms of dust levels. The process of cutting and transporting the excavated coal material leads to the formation of large amounts of harmful dust, which may have an immensely negative impact on the workers’ health once it gets into the atmosphere. At the same time, the ventilation system working in the mine makes this dust spread practically all over the mine. A substantial part of this dust also reaches the surface. As a consequence, the exposure to the harmful effects of dust occurs in practically all the areas of the mine’s underground infrastructure. Definitely the worst conditions in this regard, however, are present in the area of the longwall and dog headings located along the stream of used ventilation air. As was already pointed out, it is the ventilation air that transports the greatest amounts of dust.

The results obtained indicate that the atmospheric dust concentrations depend on both the location of the workplace and the working position occupied. Workers employed in the same positions yet in different areas of the mine are exposed to varying concentrations of dust with various content of free crystalline silica. The reason for this is that the silica content in dust depends on the mineralogical composition of the exploited seam, whereas the dust concentration levels are primarily dependent on the technical/mining factors. The highest dust levels, significantly exceeding the permissible values, were recorded mainly in the working positions directly related to the mining process.

The results obtained confirm that, despite the good recognition of the dust hazard and the application of increasingly effective measures of prevention, the exposure to the harmful effects of dust still represents a serious threat to the health of Polish workers employed in underground mine headings.

A chemical analysis of the dust present in the working positions under examination showed that, in 20% of these positions, the dust contains less than 2% of free crystalline silica, while in 80% of them—more than 2%. Significant differences were also noted in the dust concentration levels occurring within the same working position and during the performance of the same activities. Free crystalline silica has the most negative impact on the human respiratory system and determines the level of risk for the health of workers exposed to the harmful effects of dust.

Assessing the results obtained, it can be concluded that it is currently very difficult under the Polish mining/geological conditions to achieve dust concentration levels that do not exceed the MAC values, despite the application of increasingly effective measures of prevention. It is reasonable to assume that this condition will continue to negatively affect the health of workers employed in underground headings. The presented results should provide a significant source of information for the mine service departments about the actual level of dust hazards as well as encourage them to take more effective actions in order to reduce the related risk. The presented results should be used to plan preventive activities at workplaces where there is a risk of harmful dust. It should be emphasized that the problem is current and applies to the entire underground coal mining industry in Poland. In Poland, it particularly applies to employees of hard coal mines where exploitation is conducted with the use of fully powered longwalls. Similar technologies are used in other mines in the world where comparable threats occur.

The authors hope that the results obtained will be the basis of a broader discussion on the threat of dust harmful to the life and health of underground workers. This should apply to the direct protection of miners during their work underground as well as after they end working for the mining industry. A significant number of recorded cases of pneumoconiosis refers to former miners. Legislative changes also seem justified in this respect. Current regulations require to conduct measurement of the concentration of dust at workplaces at least every 12 months. Frequency of measurements seems to be insufficient, due to the changing geological and mining conditions, which have an impact on the amount of dust produced during the mining process of the body of coal. The obtained results clearly indicate that the working conditions in the mines in terms of dust hazard are still very unfavourable, and the applied solutions in the scope of limiting the dust formation are insufficient. Consequently, the threat of dust harmful to the health of Polish mine employees is a significant issue, the scale of which does not decrease. 

## Figures and Tables

**Table 1:** A summary of the average values of measurement results for dusting in the longwalls under analysis.

	Average Concentration, mg/m^3^		Standard Deviation, mg/m^3^		Median, mg/m^3^	
Longwall	Inhaled Fraction	Respirable Fraction	Inhaled Fraction	Respirable Fraction	Inhaled Fraction	Respirable Fraction
Longwall #1 (10 measurements)	7.57	3.56	2.78	1.30	6.70	3.15
Longwall #2 (10 measurements)	13.19	6.02	4.43	2.12	11.6	5.35
Longwall #3 (10 measurements)	7.14	3.32	2.31	1.13	6.80	3.10
Longwall #4 (10 measurements)	16.18	7.34	7.58	3.45	16.70	7.80
Longwall #5 (10 measurements)	20.54	9.54	4.62	2.20	22.40	10.35

**Table 2:** A summary of the average values of measurement results for crystalline silica in the dust.

Longwall	Average Concentration, %	Standard Deviation, %	Median, %
Longwall #1	2.35	0.12	2.4
Longwall #2	1.62	0.12	1.6
Longwall #3	4.94	1.03	5.2
Longwall #4	3.05	0.35	3.05
Longwall #5	3.76	0.28	3.75

**Table 3:** Limit values for dust containing free crystalline silica in compliance with Polish regulations [34].

Free Crystalline Silica Content, %	Inhalable Dust, mg/m^3^	Respirable Dust, mg/m^3^
over 50	2.0	0.3
2–50	4.0	1.0

**Table 4:** The measurement results for the dust levels for selected activities, in the working position located at the inlet of air into the longwall.

The Exploitation Phase	Dust Concentration, mg/m^3^		SiO_2_, %
	Total	Respirable	
Reaching the workplaces and preparatory works before the exploitation process	9.3	4.2	3.7
Support the main drive during cutting of the body of coal	20.1	9.7	
Mining works during a maintenance shutdown	19.5	8.9	

**Table 5:** The measurement results for the dust levels for selected activities, in the working position miner operating the scraper conveyor.

The Exploitation Phase	Dust Concentration, mg/m^3^		SiO_2_, %
	Total	Respirable	
Reaching the workplaces and preparatory works before the exploitation process	8.9	4.1	3.8
Service of scraper conveyor	24.7	11.4	
Mining works during a maintenance shutdown	21.9	10.0	

**Table 6:** The measurement results for the dust levels for selected activities, in the working position the miner working at the junction of the longwall with the tailgate (air outlet).

The Exploitation Phase	Dust Concentration, mg/m^3^		SiO_2_, %
	Total	Respirable	
Reaching the workplaces and preparatory works before the exploitation process	10.8	5.1	3.7
Moving the roof supports during cutting of the body of coal	37.7	18.1	
Mining works during a maintenance shutdown	27.3	12.9	

**Table 7:** The measurement results for the dust levels for selected activities, in the working position the miner working at a distance of 20 to 40 m from the shearer from the side of the air inlet into the longwall.

The Exploitation Phase	Dust Concentration, mg/m^3^		SiO_2_, %
	Total	Respirable	
Reaching the workplaces and preparatory works before the exploitation process	9.5	4.6	3.7
Support for auxiliary drive during cutting of the body of coal	28.7	13.4	
Mining works during a maintenance shutdown	27.0	12.6	

**Table 8:** The measurement results for the dust levels for selected activities, in the working position the cutter-loader man.

The Exploitation Phase	Dust Concentration, mg/m^3^		SiO_2_, %
	Total	Respirable	
Reaching the workplaces and preparatory works before the exploitation process	9.7	4.7	4.0
Cutting of the body of coal by mining machine	33.2	15.7	
Mining works during a maintenance shutdown	24.8	10.1	

**Table 9:** The measurement results for the dust levels for selected activities, in the working position the assistant cutter-loader man.

The Exploitation Phase	Dust Concentration, mg/m^3^		SiO_2_, %
	Total	Respirable	
Reaching the workplaces and preparatory works before the exploitation process	9.7	4.7	4.0
Auxiliary works during cutting of the body of coal	32.8	14.9	
Mining works during a maintenance shutdown	24.2	9.7	

**Table 10:** The measurement results for the dust levels for selected activities, in the working position the miner working between the shearer and the air outlet from the longwall.

The Exploitation Phase	Dust Concentration, mg/m^3^		SiO_2_, %
	Total	Respirable	
Reaching the workplaces and preparatory works before the exploitation process	10.1	4.8	4.0
Maneuvering the roof support during cutting of the body of coal	35.4	16.8	
Mining works during a maintenance shutdown	26.2	12.7	

**Table 11:** The measurement results for the dust levels for selected activities, in the working position a miner operating the belt conveyor.

The Exploitation Phase	Dust Concentration, mg/m^3^		SiO_2_, %
	Total	Respirable	
Reaching the workplaces and preparatory works before the exploitation process	8.1	3.7	3.2
Handling of conveyor and crusher	20.4	9.2	
Mining works during a maintenance shutdown	16.7	7.6	

**Table 12:** The measurement results for the dust levels for selected activities, in the working position a miner operating the dumper PZ/PT.

The Exploitation Phase	Dust Concentration, mg/m^3^		SiO_2_, %
	Total	Respirable	
Reaching the workplaces and preparatory works before the exploitation process	8.2	3.8	3.5
Operating the dumper PZ/PT, cleaning the area of exploitation	13.5	6.3	

**Table 13:** The measurement results for the dust levels for selected activities, in the working position the shift foreman.

The Exploitation Phase	Dust Concentration, mg/m^3^		SiO_2_, %
	Total	Respirable	
Reaching the workplaces and preparatory works before the exploitation process	9.5	4.6	3.6
Inspection works	28.1	13.0	
